# Supplementary material for: Antibiotic Prescribing in Dutch Daytime and Out-of-Hours General Practice during the COVID-19 Pandemic: A Retrospective Database Study
Source: Antibiotics (Basel). 2022 Feb 25;11(3):309. doi: 10.3390/antibiotics11030309 (PMC8944515; doi:10.3390/antibiotics11030309)
Supplement: Supplementary file 1 [file antibiotics-11-00309-s001.zip › antibiotics-1596037-supplementary.pdf]

Supplementary Materials

Table S1. Changes in antibiotics prescribing (total, RTIs and UTIs) between the different phases of the COVID-19 pandemic in daytime general practice and out-of-hours services, outcomes of an interrupted time series analyses.

	DGP			OOH-services		
	**Coef.**	**95% CI**	**P-value**	**Coef.**	**95% CI**	**P-value**
**Overall incidence of antibiotic prescriptions**						
Intercept	602.696	572.561,632.831	0.000	36.455	33.198,39.711	0.000
Intercept (phase 0)	-0.255	-1.115,0.604	0.560	0.009	-0.117,0.134	0.890
Intercept (phase 1)	29.095	-23.045,81.235	0.274	-0.423	-5.926,5.080	0.880
Intercept (phase 2)	149.716	49.829,249.604	0.003	3.158	-4.353,10.669	0.410
Intercept (phase 3)	121.606	17.314,225.898	0.022	-1.972	-10.832,6.888	0.663
Intercept (phase 4)	151.214	40.920,261.508	0.007	9.175	-2.893,21.243	0.136
Slope change phase 0-1	-16.315	-22.052,-10.577	0.000	-0.316	-0.651,0.019	0.065
Slope change phase 1-2	19.051	13.460,24.642	0.000	-0.001	-0.404,0.402	0.997
Slope change phase 2-3	-4.421	-6.293,-2.549	0.000	0.413	0.074,0.752	0.017
Slope change phase 3-4	5.902	4.330,7.473	0.000	-1.193	-2.801,0.416	0.146
**Incidence of antibiotic prescriptions for RTIs**						
Intercept	75.304	57.710,92.898	0.000	5.281	3.712,6.850	0.000
Intercept (phase 0)	-0.085	-0.599,0.430	0.747	0.002	-0.050,0.055	0.927
Intercept (phase 1)	28.584	7.495,49.674	0.008	2.864	0.633,5.095	0.012
Intercept (phase 2)	48.646	21.678,75.613	0.000	4.395	1.677,7.112	0.002
Intercept (phase 3)	51.277	23.887,78.668	0.000	3.910	1.117,6.703	0.006
Intercept (phase 4)	54.622	26.483,82.761	0.000	4.707	1.740,7.675	0.002
Slope change phase 0-1	-6.427	-7.730,-5.123	0.000	-0.527	-0.654,-0.400	0.000
Slope change phase 1-2	6.941	5.695,8.187	0.000	0.588	0.466,0.711	0.000
Slope change phase 2-3	-0.525	-0.883,-0.167	0.004	-0.056	-0.106,-0.005	0.032
Slope change phase 3-4	0.637	0.240,1.035	0.002	-0.109	-0.220,0.002	0.054
**Incidence of antibiotic prescriptions for UTIs**						

Intercept	106.528	103.465,109.592	0.000	9.918	8.972,10.865	0.000
Intercept (phase 0)	0.432	0.328,0.535	0.000	0.019	-0.016,0.054	0.287
Intercept (phase 1)	-15.069	-20.384,-9.754	0.000	-2.019	-3.761,-0.278	0.023
Intercept (phase 2)	-0.677	-12.175,10.821	0.908	-3.779	-6.499,-1.059	0.006
Intercept (phase 3)	-9.645	-23.473,4.183	0.172	-5.255	-8.374,-2.137	0.001
Intercept (phase 4)	-9.208	-24.784,6.368	0.247	-2.122	-6.131,1.887	0.300
Slope change phase 0-1	-1.202	-1.767,-0.636	0.000	0.206	0.051,0.360	0.009
Slope change phase 1-2	2.075	1.459,2.691	0.000	-0.215	-0.401,-0.029	0.023
Slope change phase 2-3	-2.282	-2.750,-1.813	0.000	-0.034	-0.146,0.078	0.554
Slope change phase 3-4	2.562	2.116,3.007	0.000	-0.324	-0.858,0.209	0.233

Abbreviations: CI= confidence interval; DGP=daytime general practice; OOH-services=out-of-hours services; RTI=respiratory tract infection; UTI=urinary tract infection.

Table S2. The level of antibiotic prescribing in the second intermediate phase (phase 4) of the COVID-19 pandemic in 2021 compared with the same period in 2019, for daytime general practice and out-of-hours services.

	**DGP**			**OOH-services**		
	**Mean**	**95% CI**	**P-value**	**Mean**	**95% Cl**	**P-value**
**Overall incidence of antibiotic prescriptions**						
2019 (same period as phase 4)	560.031	554.287 - 565.776	<0.001	39.184	34.021 - 44.346	0.314
2021 (phase 4)	510.815	494.751 - 526.880		36.610	34.164 - 39.056	
**Incidence of antibiotic prescriptions for RTIs**						
2019 (same period as phase 4)	48.092	43.234 - 52.950	<0.001	3.998	3.205 - 4.790	0.004
2021 (phase 4)	32.100	29.411 - 34.789		2.747	2.462 - 3.031	
**Incidence of antibiotic prescriptions for UTIs**						
2019 (same period as phase 4)	120.727	116.283 – 125.171	0.151	10.694	9.426 - 11.962	0.794
2021 (phase 4)	125.596	120.342 – 130.850		10.871	9.999 - 11.743	

Abbreviations: CI=confidence interval; DGP=daytime general practice; OOH-services=out-of-hours services; RTI=respiratory tract infection; UTI=urinary tract infection.
